# The first complete mitochondrial genome in the family Attevidae (*Attevaaurea*) of the order Lepidoptera

**DOI:** 10.3897/BDJ.10.e89982

**Published:** 2022-09-12

**Authors:** Jun Seong Jeong, Jeong Sun Park, Jae-Cheon Sohn, Min Jee Kim, Hyung Keun Oh, Iksoo Kim

**Affiliations:** 1 Division of Genetic Resources, Honam National Institute of Biological Resources, Mokpo, Republic of Korea Division of Genetic Resources, Honam National Institute of Biological Resources Mokpo Republic of Korea; 2 Department of Applied Biology, College of Agriculture & Life Sciences, Chonnam National University, Gwangju, Republic of Korea Department of Applied Biology, College of Agriculture & Life Sciences, Chonnam National University Gwangju Republic of Korea; 3 Department of Science Education, Gongju National University of Education, Chungnam, Republic of Korea Department of Science Education, Gongju National University of Education Chungnam Republic of Korea; 4 Experiment and Analysis Division, Honam Regional Office, Animal and Plant Quarantine Agency, Gunsan, Republic of Korea Experiment and Analysis Division, Honam Regional Office, Animal and Plant Quarantine Agency Gunsan Republic of Korea; 5 Crop Protection Team, Chamfield Co., Ltd, Hanam, Republic of Korea Crop Protection Team, Chamfield Co., Ltd Hanam Republic of Korea

**Keywords:** mitochondrial genome, *Attevaaurea*, phylogeny, Attevidae

## Abstract

The superfamily Yponomeutoidea, one of the early-derived groups in the order Lepidoptera, consists of 11 families. However, mitochondrial genome (mitogenome) sequences, popularly used for phylogeny and evolutionary tracing, are available for only seven species across six genera and five families. Thus, a larger variety of mitogenome sequences in Yponomeutoidea are required to improve our understanding of lepidopteran phylogeny and genomic evolution. In this study, we present the complete mitogenome of *Attevaaurea* (Fitch, 1856), the first species in the family Attevidae (superfamily Yponomeutoidea, order Lepidoptera) to be sequenced. The complete mitogenome comprises 16,329 bp and contains a typical set of genes and one non-coding region. Within Yponomeutoidea, the mitogenome of *A.aurea* has a unique *trnI*-*trnM*-*trnQ* arrangement at the A + T-rich region and *ND2* junction and *trnA*-*ND3* arrangement at the *trnG* and *trnR* junction. Twelve of the 13 protein-coding genes (PCGs) of *A.aurea* have a typical ATN starting codon, whereas *COI* has the atypical CGA codon, which is frequently found in the starting region of lepidopteran *COI*. Phylogenetic analyses, based on the concatenated sequences of 13 PCGs and two rRNA genes, using the Maximum Likelihood method, revealed a sister relationship between Attevidae and Praydidae with moderately low nodal support (bootstrap support = 64%).

## Introduction

The superfamily Yponomeutoidea is one of the earliest groups to develop external feeding mechanisms in the order Lepidoptera and comprises ~ 1,800 species across 11 families (Sohn et al. 2013). However, only seven species in six genera across five families have available mitochondrial genome (mitogenome) sequences. Thus, the characterisation of the mitogenomes of more families will significantly contribute to the study of genomic evolution and subsequent phylogenetic analysis within this superfamily, as well as other early-derived lepidopteran clades.

The ailanthus webworm (*Attevaaurea* Fitch, 1856) is a small, colourful moth predominantly found north of Costa Rica, across the USA and in southern Quebec and Ontario, Canada (Wilson et al. 2010). Populations distributed south of Costa Rica in Uruguay and Argentina are known as *A.pustulella* (Fabricius, 1787), the former classification of *A.aurea* in North America (Wilson et al. 2010).

In this study, we present the complete mitogenome of *A.aurea*, the first species in the family Attevidae (superfamily Yponomeutoidea, order Lepidoptera) to be sequenced. The sequence was analysed in terms of its mitogenome characteristics and phylogenetic position within the superfamily Yponomeutoidea. Additionally, the DNA barcoding region of *A.aurea* was compared to that of previously-registered *A.aurea* and *A.pustulella*, which have been used for extensive phylogenetic analysis (Wilson et al. 2010), to further confirm sequence divergence between the two species.

## Materials and methods

### Sample collection, DNA extraction, PCR and sequencing

In 2011, a brood of *A.aurea* was collected from the Paint Branch Trail at the University of Maryland (College Park, MA, USA; 38°59’39’’N, 76°56’5’’W). In this study, DNA was extracted from the whole body of one adult male using the Wizard Genomic DNA Purification Kit (Promega, Madison, WI, USA). Using Lepidoptera-specific primers (Suppl. material 1, Kim et al. 2012), three overlapping long fragments (LFs; *COI* to *ND4*, *ND5* to *lrRNA* and *lrRNA* to *COI*) were amplified. These LFs were then used as templates for the amplification of 26 short fragments (SFs) using the same Lepidoptera-specific primers (Suppl. material 1, Kim et al. 2012). All products were sequenced in both forward and reverse transcriptional directions by Sanger's methods. The whole body of the specimen was consumed in the process. Thus, other individuals of the brood were moved as voucher specimens to the Gongju National University of Education (Gongju, South Korea) and labelled with accession nos. GNUE-I-0001–GNUE-I-0003.

### Boundary delimitation and annotation

Individual SF sequences were manually assembled into complete mitogenomes using SeqMan (DNASTAR, Madison, WI, USA). The identification and boundary delimitation of each gene and secondary structure folding of tRNAs were performed using the MITOS Web Server (http://mitos.bioinf.uni-leipzig.de/index.py) and using the default search mode, Mito/Chloroplast as the searching source and the genetic code of invertebrate mitogenomes for tRNA isotype prediction (Lowe and Chan 2016). Where necessary, mitogenome sequences of species in the superfamily Yponomeutoidea registered in GenBank were downloaded and aligned for improved annotation by following the protocols presented by Cameron 2014.

### Phylogenetic analysis

Phylogenetic analysis was conducted using 25 available mitogenomes in 23 species (including *A.aurea*) in the superfamilies Gracillarioidea, Yponomeutoidea and Tineoidea. We selected Gracillarioidea and Tineoidea, along with Yponomeutoidea, because of the previously established sister-group relationship between Yponomeutoidea and Gracillarioidea and of the branching of Tineoidea as a lineage basal to these two superfamilies (Timmermans et al. 2014, Breinholt et al. 2018, Bao et al. 2019, *Kawahara et al. 2019*). Two species within the superfamily Nepticuloidea (*Stigmellaroborella* and *Astrotischeria* sp.) were used as outgroups. Thirteen protein-coding genes (PCGs) and two rRNA genes (including those of two outgroup species) were aligned using RevTrans ver. 2.0 (Wernersson and Pedersen 2003) and concatenated using SequenceMatrix ver. 1.8 (Vaidya et al. 2011). The Maximum Likelihood method was applied using CIPRES Portal ver. 3.1 (Miller et al. 2010) for phylogenetic analyses, based on the GTR + Gamma + I model, which was selected using jModelTest (Posada 2008).

## Data resources

Genome sequence data used in this study are openly available from the GenBank database of the National Center for Biotechnology Information (https://www.ncbi.nlm.nih.gov) under the accession no. ON480203. All datasets used in this study were published at Zenodo on 03 July 2022 (Zenodo. https://doi.org/10.5281/zenodo.6791899).

## Results and discussion

### Genome summary

The complete 16,392-bp mitogenome of *A.aurea* is composed of typical gene sets (two rRNAs, 22 tRNAs and 13 PCGs) and a major non-coding A + T-rich region (Table 1; GenBank accession no. ON480203). Twelve of the 13 PCGs have the typical ATN start codon, whereas *COI* has the atypical CGA codon, which is found in the majority of other available mitogenome sequences of Tineoidea, Gracillarioidea and Yponomeutoidea species (data not shown), as well as Lepidoptera species (Kim et al. 2010, Park et al. 2016, Kim et al. 2018, Jeong et al. 2021). Ten PCGs end with typical stop codon TAA, whereas *COII* and *ND4* have incomplete, single-thymine stop codons. The A/T content is 79.9% in PCGs, 82.4% in tRNAs, 82.5% in the whole genome, 86.5% in *lrRNA*, 87.6% in *srRNA* and 98.0% in the A + T-rich region (data not shown).

**Figure 1.**
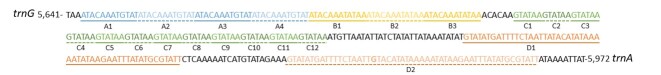
Four tandem repeat units found between *trnG* and *trnA* with various copy numbers (A1–A4, B1–B3, C1–C12 and D1–D2). The nucleotide position is indicated at each end of the sequence in relation to the mitochondrial genome of *Attevaaurea*.

The genes of *A.aurea* are interleaved with a total 627 bp, spread over 17 regions ranging in size between 1 and 332 bp (Table 1). Most intergenic spacer sequences (ISSs) are short (1–20 bp), but four locations have longer ISSs (41–332 bp). Examination with the naked eye revealed that three of those four ISSs (*trnQ*-*ND2*, *COI*- *trnL_2_* and *trnA*-*ND3*) have no notable features, except high A/T content (90.24–96.30%; data not shown). However, the longest of these ISSs (332 bp), located between *trnG* and *trnA*, has four tandem repeat units with varying copy numbers (Fig. 1; A1–A4, B1–B3, C1–C12 and D1–D2). All copies of each repeat unit have identical sequences, except for one nucleotide substitution (A for G) in repeat unit D.

### Gene rearrangement

**Figure 2.**
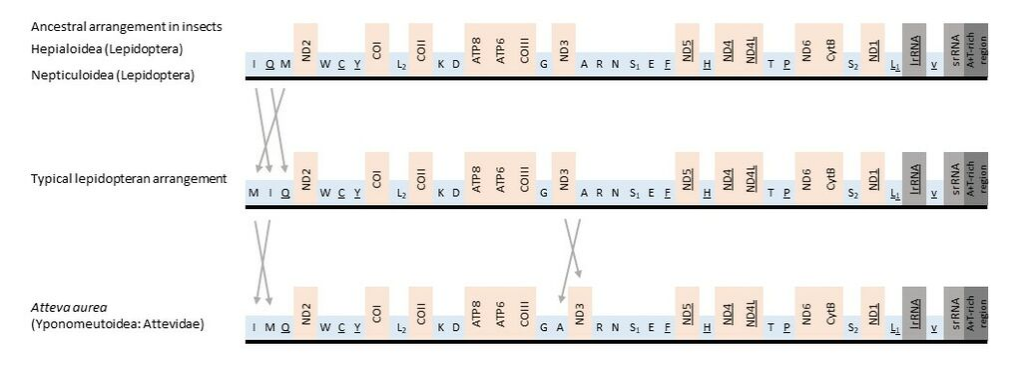
Linear arrangement of the mitochondrial genome of *A.aurea*. Gene sizes are not drawn to scale. Non-underlined and underlined gene names indicate forward and reverse transcriptional directions, respectively. Translocated genes are indicated by lines with arrows.

Compared with that of other Lepidoptera species, the *A.aurea* mitogenome has a very rare *trnI*-*trnM*-*trnQ* arrangement (underlining indicates gene inversion) at the A + T-rich region and *ND2* junction (Fig. 2). *Monopislongella* (Walker, 1863) (family Tineidae, superfamily Tineoidea) is the only species previously known to exhibit the *trnI*-*trnM*-*trnQ* arrangement in the Ditrysia clade, including the superfamilies Gracillarioidea, Yponomeutoidea, and Tineoidea (Jeong et al. 2021). Conversely, the majority of ditrysian Lepidoptera species have the gene order *trnM*-*trnI*-*trnQ* at the same junction (Fig. 2, Kim et al. 2014). This differs from the ancestral *trnI*-*trnQ*-*trnM* order found in most insects (Fig. 2, Boore 1999), including ancient, non-ditrysian lepidopteran groups, such as Hepialoidea and Nepticuloidea (Cao et al. 2012, Timmermans et al. 2014). Moreover, the *A.aurea* mitogenome has the *trnA*-*ND3* arrangement at the *trnG* and *trnR* junctions instead of the *ND3*-*trnA* arrangement found in almost all Lepidoptera species, including all those in Gracillarioidea, Yponomeutoidea, and Tineoidea (Fig. 2, Kim et al. 2014, Jeong et al. 2021). Thus far, only seven species in six genera across fiv families in Yponomeutoidea have had their mitogenomes sequenced. Thus, further analysis of this superfamily is required to make any conclusive remarks about the evolution of this rearrangement. Nevertheless, current analysis indicates that the arrangement of the family Attevidae is an autapomorphic characteristic of the superfamily Yponomeutoidea (data not shown).

### Comparison of DNA barcoding sequence

The comparison between the DNA barcoding sequences of current *A.aurea* and those of *A.aurea* previously registered on GenBank, including those registered by Wilson et al. (2010), showed a 0.00–1.67% divergence. Compared with *A.pustulella* DNA barcoding sequences, there was a divergence of at least 3.95% (data not shown). This reflects the findings of a previous study that *A.aurea*, distributed between Costa Rica and southern Quebec and Ontario, is indeed *A.aurea* and that phylogenetic results demonstrate a clear separation of *A.aurea* from other Attevidae species, including *A.pustulella* (Wilson et al. 2010).

### Phylogenetic analysis

**Figure 3.**
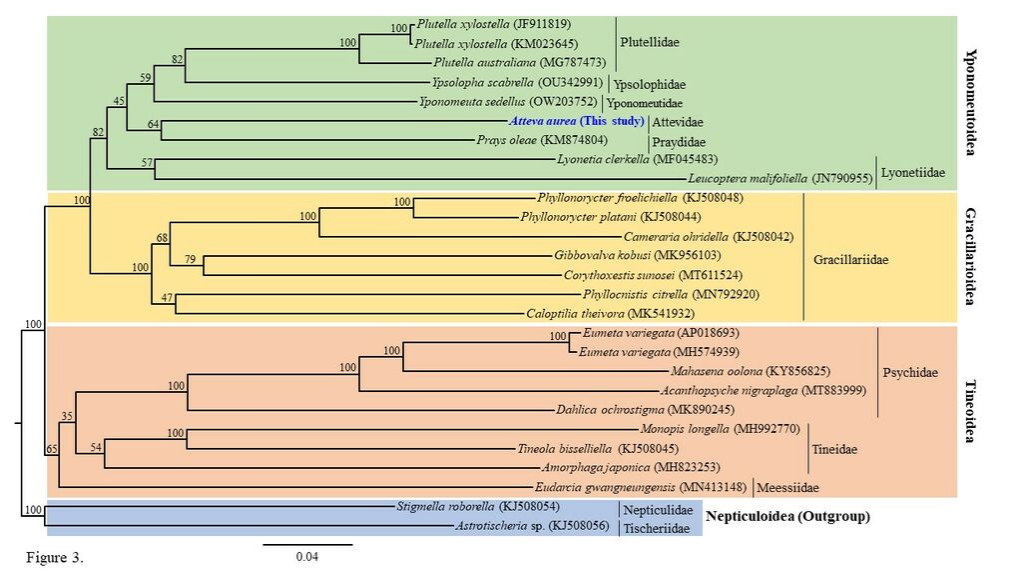
Phylogenetic tree of the three ditrysian superfamilies included in this study: Tineoidea, Gracillarioidea and Yponomeutoidea. The number at each node indicates the bootstrap value. The scale bar indicates the number of substitutions per site. The GenBank accession number of each species is shown in brackets after its scientific name.

Phylogenetic analysis revealed overall lower nodal supports for familial relationships within Yponomeutoidea. A sister relationship between the families Attevidae and Praydidae, each of which is represented by a single species, was supported, but the nodal support for this relationship was not high (bootstrap support (BS) = 64%; Fig. 3). Within the Ditrysia clade, Gracillarioidea and Yponomeutoidea exhibit a sister relationship with the highest nodal support, placing Tineoidea sister to the two superfamilies with the highest support (Fig. 3). Previously, Sohn et al. (2013), using 8.0–18.9 kb of 8–27 genes from 11 families in Yponomeutoidea, also revealed a sister relationship between Attevidae and Praydidae and this relationship was supported with relatively high nodal support. In terms of relationships between superfamilies, our findings are consistent with previous studies based on mitogenomic, molecular, morphological, genomic and transcriptome data that proposed a sister-group relationship between Yponomeutoidea and Gracillarioidea, with Tineoidea diverging earlier within ditrysian Lepidoptera (Heikkilä et al. 2014, Timmermans et al. 2014, Breinholt et al. 2018, Bao et al. 2019, Kawahara et al. 2019). Additional phylogenetic relationships within the early-derived groups of Lepidoptera could be determined, based on further taxonomic research with a wider scope.

## Conclusions

This mitogenome of *A.aurea* has a unique *trnI*-*trnM*-*trnQ* arrangement at the A + T-rich region and *ND2* junction and *trnA*-*ND3* arrangement at the *trnG* and *trnR* junction, which is unprecedented in Yponomeutoidea. Thus, additional mitogenome sequences are required from other genera, subfamilies and families to understand the taxonomic extent of this arrangement in Yponomeutoidea. Phylogenetic analysis revealed a sister relationship between Attevidae and Praydidae, consistent with the results of a previous large-scale molecular phylogenetic study, but nodal support was not high in this study. The result of our DNA barcoding sequence comparison supports the finding of a previous study that *A.aurea*, occurring north of Costa Rica in the USA and southern Quebec and Ontario, is genetically distinct from *A.pustulella* distributed from Costa Rica south to Uruguay and Argentina. Including that of *A.aurea*, only nine mitogenome sequences, representing seven genera across six families, are currently available for the superfamily Yponomeutoidea. Thus, more mitogenome sequences from the early-derived groups of Lepidoptera, including Yponomeutoidea, are essential for a greater understanding of mitogenome evolution and phylogenetic relationships in this order.

## Supplementary Material

9982D6C3-9846-5B76-9F71-32377B07A191 10.3897/BDJ.10.e89982.suppl1 Supplementary material 1 List of primers used to amplify and sequence the *Attevaaurea* mitochondrial genome Data type Primer list File: oo_710151.docx https://binary.pensoft.net/file/710151 Jeong et al.

## Figures and Tables

**Table 1.** Summary of *Attevaaurea* mitochondrial genome.

Gene	Nucleotide number	Size	Anticodon	Start codon	Stop codon	O/S
*trnI*	1-64	64	GAT 29-31			
*trnM*	65-132	68	CAT 96-98			-8
*trnQ*	141-209	69	TTG 177-179			-41
*ND2*	251-1276	1026		ATT	TAA	+2
*trnW*	1275-1340	64	TCA 1305-1307			+8
*trnC*	1333-1401	69	GCA 1369-1371			-7
*trnY*	1409-1474	66	GTA 1441-1443			-5
*COI*	1480-3015	1536		CGA	TAA	-54
*trnL_2_*	3070-3136	67	TAA 3100-3102			
*COII*	3137-3818	682		ATG	T-tRNA	
*trnK*	3819-3889	71	CTT 3849-3851			+1
*trnD*	3889-3954	66	GTC 3919-3921			
*ATP8*	3955-4116	162		ATC	TAA	+7
*ATP6*	4110-4781	672		ATG	TAA	+1
*COIII*	4781-5569	789		ATG	TAA	-2
*trnG*	5572-5640	69	TCC 5602-5604			-332
*trnA*	5973-6039	67	TGC 6002-6004			-103
*ND3*	6143-6517	375		ATA	TAA	-28
*trnR*	6546-6607	62	TCG 6573-6575			-4
*trnN*	6612-6677	66	GTT 6642-6644			+1
*trnS_1_*	6677-6737	61	GCT 6698-6700			-23
*trnE*	6761-6826	66	TTC 6791-6793			+2
*trnF*	6825-6892	68	GAA 6856-6858			-3
*ND5*	6896-8629	1716		ATT	TAA	
*trnH*	8630-8694	65	GTG 8659-8661			
*ND4*	8695-10036	1342		ATG	T-tRNA	+1
*ND4L*	10036-10323	288		ATG	TAA	-2
*trnT*	10326-10390	65	TGT 10357-10359			
*trnP*	10391-10455	65	TGG 10424-10426			-1
*ND6*	10457-10981	525		ATT	TAA	-3
*CytB*	10985-12136	1152		ATG	TAA	-10
*trnS_2_*	12147-12215	69	TGA 12179-12181			+2
*ND1*	12214-13170	957		ATG	TAA	-1
*trnL_1_*	13172-13238	67	TAG 13207-13209			
*lrRNA*	13239-14559	1321				
*trnV*	14560-14625	66	TAC 14594-14596			
*srRNA*	14626-15393	768				
A + T–rich region	15394-16392	999				
Non-underlined and underlined genes indicate forward and reverse transcriptional directions, respectively. tRNAs are denoted as one-letter symbols in accordance with the IUPAC-IUB single-letter amino acid codes, except those encoding leucine and serine, which are labelled *L_1_* for the CTN, *L_2_* for the TTR, *S_1_* for the AGN and *S_2_* for the TCN codon families. O/S denotes the number of the overlapping(+)/intergenic space sequence(-).						
