# Supplementary material for: The first complete mitochondrial genome in the family Attevidae (Attevaaurea) of the order Lepidoptera
Source: Biodivers Data J. 2022 Sep 12;10:e89982. doi: 10.3897/BDJ.10.e89982 (PMC9848453; doi:10.3897/BDJ.10.e89982)
Supplement: Supplementary material 1 — List of primers used to amplify and sequence the Attevaaurea mitochondrial genome [file bdj-10-e89982-s001.docx]

**Supplementary material 1.** List of primers used to amplify and sequence the *Atteva aurea* mitochondrial genome.

| Fragment name | | Primer name | Direction^a^ | Sequence (5’- 3’) | Nucleotide position^b^ | Mismatch^c^ |
| --- | --- | --- | --- | --- | --- | --- |
| Long fragments | | |  |  |  |  |
| LF1 | Lep-COI-F1 | | F | TTCTACAAATCATAAAGATATTGG | 1493-1516 | 1 |
|  | Lep-ND4-R1 | | R | ATTGGTCATGGTTTATGTTCTTC | 9099-9121 | 1 |
|  |  | |  |  |  |  |
| LF2 | Lep-ND5-F1 | | F | CTAAAAGGAATTTGAGCTCT | 8025-8044 | 1 |
| 〃 | Lep-lrRNA-R2 | | R | GTATCTTGTGTATCAGAGTTTA | 14373-14394 | 1 |
|  |  | |  |  |  |  |
| LF3 | Lep-lrRNA-F1 | | F | TGTAAGATTTTAATGATCGAACAGAT | 13366-13391 | 0 |
| 〃 | Lep-COI-R1 | | R | CTTCAGGATGACCAAAAAATC | 2177-2197 | 1 |
|  |  | |  |  |  |  |
| Short fragments | | |  |  |  |  |
| SF1 | YP-SF1-F1 | | F | TAAATTAAGCTTTTGGGTTC | 77-96 | 1 |
| 〃 | AA-SF1-R1 | | R | ATTTTCTCTAATTATAATGGC | 821-841 | 0 |
|  |  | |  |  |  |  |
| SF2 | LF03-S06-F2 | | F | ATTRTWGAAGGWTTATCWTG | 605-624 | 0 |
| 〃 | AA-SF2-R1 | | R | ATTCTATTTAAGATATAAAATTGC | 1370-1393 | 0 |
|  |  | |  |  |  |  |
| SF3 | LF03-S07-F1 | | F | AATCTTCAAAATTATTTATAAAG | 1300-1322 | 2 |
| 〃 | Lep-COI-R1 | | R | CTTCAGGATGACCAAAAAATC | 2177-2197 | 1 |
|  |  | |  |  |  |  |
| SF4 | LF01-S01-F2 | | F | TTACAACAATTATTAATATACG | 1964-1985 | 1 |
| 〃 | LF01-S01-R2 | | R | GTCGAGGTATTCCTGCTA | 2768-2785 | 1 |
|  |  | |  |  |  |  |
| SF5 | LF01-S02-F1 | | F | AATTTTTAGTTGATTAGCWAC | 2427-2447 | 0 |
| 〃 | LF01-S02-R1 | | R | GATCATGAAAAAAAATAATTTG | 3191-3212 | 0 |
|  |  | |  |  |  |  |
| SF6 | LF01-S03-F2 | | F | TAGAAATGGCAACWTGATC | 3132-3150 | 0 |
| 〃 | LF01-S03-R1 | | R | CTTGCTTTCAGTCATCTAAT | 3820-3839 | 1 |
|  |  | |  |  |  |  |
| SF7 | AA-LF7-F2 | | F | TCATAGTTTTATACCTATTG | 3745-3764 | 0 |
| 〃 | AA-SF7-R1 | | R | TAATTAAATGACTTGTTCTAG | 4387-4407 | 0 |
|  |  | |  |  |  |  |
| SF8 | LF01-S05-F2 | | F | TTTTATTTAATAATTTTTTAGG | 4345-4366 | 1 |
| 〃 | LF01-S05-R1 | | R | CTCGTCATCATTGATATAT | 4943-4961 | 0 |
|  |  | |  |  |  |  |
| SF9 | LF01-S06-F1 | | F | CWGTWGCAATTATTCAATC | 4714-4732 | 1 |
| 〃 | LF01-S06-R1 | | R | GTCAATATCAAGCWGCTG | 5493-5510 | 1 |
|  |  | |  |  |  |  |
| SF10 | AA-SF10-F4 | | F | ACTTATTTGTTTAATTCGAC | 5428-5447 | 0 |
| 〃 | AA-SF10-R3 | | R | TTCAATTTTATCATTAACAATG | 6638-6659 | 0 |
|  |  | |  |  |  |  |
| SF11 | LF01-S08-F1 | | F | TAGAAATTGCATTAATTTTHCC | 6346-6367 | 2 |
| 〃 | AA-LF1-R1 | | R | GATTACATATAAAACTTATGG | 7252-7272 | 0 |
|  |  | |  |  |  |  |
| SF12 | LF01-S09-F1 | | F | AWAHTTCTCTTCAACCYAWATC | 7036-7057 | 2 |
| 〃 | LF01-S09-R1 | | R | CATTTATTAACTCATGCTAT | 7716-7735 | 0 |
|  |  | |  |  |  |  |
| SF13-14 | AA-SF13-F2 | | F | AAATCGAATATCCTGTGTATC | 7643-7663 | 0 |
| 〃 | AA-SF14-R1 | | R | CATTGATTWCCTTTAAATAT | 8733-8752 | 0 |
|  |  | |  |  |  |  |
| SF15 | LF01-S12-F1 | | F | ATATTTTTGAYHCCACAAATC | 8649-8669 | 1 |
| 〃 | LF01-S12-R2 | | R | CAGGTTCAATAATTTTAGC | 9375-9393 | 2 |
|  |  | |  |  |  |  |
| SF16 | LF02-S01-F2 | | F | TGAGCWACWGAAGAATAAGC | 9192-9211 | 0 |
| 〃 | LF02-S01-R1 | | R | GGTTTAATTTTATTAAGAATTTG | 9834-9856 | 1 |
|  |  | |  |  |  |  |
| SF17 | AA-SF17-F2 | | F | ATATTAAAGTAGGAATTAAWC | 9659-9679 | 0 |
| 〃 | AA-SF17-R1 | | R | TAATTTTGGAGATTATWGAT | 10413-10432 | 0 |
|  |  | |  |  |  |  |
| SF18 | LF02-S03-F1 | | F | CCTAAAGCHCCYTCACAAAC | 10106-10125 | 1 |
| 〃 | LF02-S03-R1 | | R | GTAATTTTTACWACTGCAATTA | 10920-10941 | 3 |
|  |  | |  |  |  |  |
| SF19 | LF02-S04-F1 | | F | TTAAWACATATTGATTTTCTTA | 10589-10610 | 1 |
| 〃 | LF02-S04-R1 | | R | GATATTTGTCCYCAAGGTA | 11394-11412 | 2 |
|  |  | |  |  |  |  |
| SF20 | LF02-S05-F1 | | F | TAAATTATGGWTGATTAATTCG | 11214-11235 | 2 |
| 〃 | LF02-S05-R1 | | R | CCTTGDATTTTTTTATTAAADGT | 11915-11937 | 2 |
|  |  | |  |  |  |  |
| SF21 | LF02-S06-F1 | | F | TTACTTTTAAAGATTTAATTGG | 11668-11689 | 2 |
| 〃 | LF02-S06-R1 | | R | GAGAATTAGTTTCAGGRTTTA | 12513-12533 | 1 |
|  |  | |  |  |  |  |
| SF22 | LF02-S07-F2 | | F | AAAGCAAATCCCCCTCTTC | 12483-12501 | 1 |
| 〃 | LF02-S07-R2 | | R | CTGAGTTCAAACCGGTGTRA | 13342-13361 | 1 |
|  |  | |  |  |  |  |
| SF23 | LF02-S08-F2 | | F | GAHTTCTAAAAYCATTAC | 13202-13219 | 0 |
| 〃 | LF02-S08-R1 | | R | GACTGTACAAAGGTAGCATAAT | 13791-13812 | 0 |
|  |  | |  |  |  |  |
| SF23.5 | AA-SF23.5-F2 | | F | TCCAATCATTCATACCAGTC | 13752-13771 | 0 |
| 〃 | AA-SF23.5-R1 | | R | CCTGTTTATTAAAAACATGTC | 13876-13896 | 0 |
|  |  | |  |  |  |  |
| SF24 | LF03-S02-F1 | | F | ATTATGCTACCTTTGTACAGTC | 13791-13812 | 1 |
| 〃 | LF03-S02-R1 | | R | GTATTTCATTTACATTGAAAAGA | 14584-14606 | 1 |
|  |  | |  |  |  |  |
| SF25 | LF03-S03-F3 | | F | CTCTGATACACAAGATAC | 14377-14394 | 1 |
| 〃 | LF03-S03-R3 | | R | CCAGCAGTTGCGGTTAAAC | 15214-15232 | 1 |
|  |  | |  |  |  |  |
| SF26 | AA-SF26-F2 | | F | AATAGGGTATCTAATCCTAG | 15060-15079 | 0 |
| 〃 | AA-SF26-R1 | | R | ATATTTTAGTGTAAGATGCAC | 188-208 | 0 |

^a^ F and R denote forward and reverse transcriptional directions, respectively.

^b, c^ Nucleotide positions and mismatches, respectively, with respect to the *Atteva aurea* mitochondrial genome.
